# Obstetric referrals to a tertiary care maternity: a descriptive study

**DOI:** 10.11604/pamj.2019.33.306.16906

**Published:** 2019-08-19

**Authors:** Mariem Ghardallou, Manel Limam, Abdejelil Khelifi, Ons Khairi, Hédi Khairi, Ali Mtiraoui, Thouraya NabliAjmi

**Affiliations:** 1 Université de Sousse, Faculté de Médecine de Sousse, Département de Médecine Communautaire et Familiale, 4000, Sousse, Tunisie; 2 Université de Sousse, Faculté de Médecine de Sousse, Laboratoire de Recherche en Qualité des Soins Maternelles, “LR12ES03”, 4000, Sousse, Tunisie; 3 Université de Sousse, Faculté de Médecine de Sousse, Département d'Obstétrique et de Gynécologie, Hôpital Universitaire Farhat Hached, 4000, Sousse, Tunisie

**Keywords:** Referral system, obstetric, reasons, diagnoses

## Abstract

**Introduction:**

An effective referral system is considered as a key to saving mothers' and children's lives. The aims of this study were to determine the frequency and the indications of obstetric referrals in a Tunisian tertiary care maternity and to assess the conformity of referral mechanisms with the National Perinatality Programme (NPP) guidelines.

**Methods:**

A descriptive study was undertaken among women referred to Farhat Hached University Hospital in Sousse, Tunisia with antenatal complications requiring urgent delivery and those referred while in labour or with immediate post partum complications. The ICD-10 was used to code recorded indications and diagnoses for referrals.

**Results:**

Referrals represents 15.23% of the obstetric activity in this facility. There were 32 reasons for referrals with the most common being premature rupture of membranes (14.1%) and fetal distress (13.5%). A fifth of the referrals were unclassifiable according to ICD-10. Most of the indications for referrals (95.8%) did not conform to the list of referral indications of the NPP. Twenty eight diagnoses were retained after referrals: the most common of which were prolonged pregnancy (29.5%) and premature rupture of membranes (19.3%). In 41% of women, reasons for referral did not match with diagnoses established at the time of the patients' admission to hospital.

**Conclusion:**

The current referral system in the region of Sousse still faces several challenges that need to be addressed in order to make it more effective.

## Introduction

For decades, improving maternal health has been a worldwide priority component of global health and development community [1]. In fact, in the 2000 Millennium summit, reducing the Maternal Mortality Ratio (MMR) by 75% between 1990 and 2015 was the fifth Millennium Development Goal (MDG5) [2]. Despite efforts towards attainment MDG5, Maternal Mortality (MM) is still at high levels especially in low and middle income countries such as Tunisia. In fact, to achieve MDG5, the Tunisian government launched the National Safe Motherhood initiative in 1999 [1] that was assessed via the Reproductive Age Mortality Study (RAMOS) methodology. A sharp decrease in the MMR was noted with a drop from 68.9 per 100 000 live births in 1993-1994 [1] to 36.3 per 100 000 live births in 2005-2007. According to the National Committee on Maternal Death, about 85% of direct obstetric deaths were classified as preventable or conditionally preventable [1] and were to a great extent, related to health care system factors such as the shortage of material (drugs, blood…) or human resources and the inadequacy of the existing referral system. In fact, access to all levels of care system is considered as a key dimension in saving mothers' live [3]. “Referral” means “any upwards movement of health care seeking individuals in the health system” [4]. There are many ways to do this with respect to pathway, timing and urgency. So, it will be designed-"Evacuation" when it is urgent [4, 5]. A formalised maternity referral system lie within strategy of risk screening in the antenatal period, in which frontline health workers would attempt to identify those women at high risk of obstetric complications and refer them on for specialized antenatal and delivery care at a higher level [3].

According to the WHO maternal health and safe motherhood program estimates, obstetric references account for more than 20% of pregnancy-related morbidity [6] and according to previous African studies, obstetric evacuations from peripheral health structures represent 3 to 66% of the activities of the receiving hospitals and are associated with a high maternal and fetal morbi-mortality [7]. In Tunisia, inadequate obstetric transfer was responsible for almost half of maternal deaths (48.38%) between 1998 and 2007 in a third level maternity in Tunis (Tunisia) [8]. Rapid screening and transfer in good conditions of pregnant women at risk would theoretically reduce maternal mortality rate by half [8]. For a more effective planning of medical and community-based actions for both mother and child, we have to assess this system of referral. Thus, we conducted this study aiming to determine the frequency and the epidemiological profile of obstetric referrals in a Tunisian tertiary care maternity, and identifying the major indications for such transfer during labour and delivery periods and to assess the conformity of referral mechanisms with the guidelines of the National Perinatality Programme.

## Methods

**Design and Setting:** a cross sectional descriptive study was conducted in a tertiary care maternity in Sousse (Tunisia) over a period of two months from 1^st^ September to 30^th^ November 2014. This institution is a public medical care center and one of the most important maternal care facilities in Tunisia. It receives referrals mainly from the lower level facilities (peripheral maternities) within the Sousse Health region, but also within the surrounding areas. This maternity is part of the Department of Obstetrics and Gynecology of Farhat Hached (FH) University Hospital [9]. In most of these peripheral maternities, the pregnant women are cared for almost solely by midwives [10]. The annual number of deliveries in the region of Sousse was about 12 261 in 2013, of which 10 169 (82.9%) occurred in FH maternity [11].

**Population:** we included all women referred with pregnancy related complications while pregnant (antenatal complications needing urgent delivery), during labour or within immediate post partum. It was an exhaustive sample from all women referred to the center during the period of study.

**Data collection and analysis:** after delivery or during post-natal hospitalisation, the following informations were extracted from medical records: current pregnancy characteristics, management of childbirth in the referring maternity, maternal and foetal outcomes. Further data on the first clinical examination done in the referring maternity and the indications for referral were extracted from the referral letters. Moreover, women were interviewed after delivery about socio-demographic characteristics, past obstetric history, conditions of referral (means of transport by which they came to the hospital, waiting times, staff accompanying to hospital). For more precision, informations about the final diagnoses and prognoses were obtained from the receiving midwife at the FH maternity. The International Statistical Classification of Diseases (ICD-10) and related health problems were used to code recorded indications and diagnoses for referral. Descriptive data analysis was performed using SPSS. Quantitative variables were presented as means with standard deviation when they were normally distributed. Qualitative variables were described as frequencies and percentages.

**Ethical considerations:** a verbal consent was obtained from all participants and all information was treated in confidentiality.

## Results

During the period of study, there were 513 referrals, out of 3367 obstetric admissions which represent 15.23% of the obstetric activity in this facility. Only 500 cases of referrals were considered for this study. The mean age of the referred women was 29.5±5.6 years. More than a half of them (57.8%) were from rural areas and about 86% of them did not study beyond secondary level of education. The median of parity was 2, with a parity distribution as follows: nulliparous: 238 (47.6%); primiparious: 114 (22.8%), multiparious: 140 (28%) and above 5 parity: 8 (1.6%). About all women (98.4%) had an inter-pregnancy interval (IPI) ≥2 years. Only three women had had perinatal deaths, thirty (6%) had history of sterility and 63 (12.7%) had caesarean sections. The majority of women (98.6%) had followed the progress of their pregnancy, and 81.6% on average, had received adequate prenatal care (≥4 antenatal visits). This prenatal follow-up was done in 89.5% of cases by a specialist in obstetrics. The majority of cases (74.7%) had no adverse factors in their current pregnancy.

**Sources and reasons for referral:** a total of 390 women (78.1%) were referred from public peripheral maternities of the region of Sousse, 21.8% from maternities outside Sousse and only one woman was referred from a private clinic in Sousse. There were 32 reasons for referral (1.6 reason ranging from 0 to 6 reasons per woman) and the commonest indications were premature rupture of membranes (14.1%) and foetal distress (13.5%). A fifth (20.8%) of indications of referral was unclassifiable according to ICD-10 (Table 1). Most of the indications for referral (95.8%), such as clear amniotic liquid, history of big baby, low maternal age, elderly primgravid…, did not conform to the list of referral indications of the National Perinatality Programme.

**Table 1:** Reasons for referral

	*Reasons for referral*	*N*	*%*
1	Premature rupture of membranes	110	14.1
2	Labour and delivery complicated by fetal stress	105	13.5
3	Maternal care for other Diseases	82	10.5
4	Maternal care for abnormality of pelvic organs	65	8.3
5	Prolonged pregnancy	42	5.4
6	Abnormal findings on antenatal screening of mother	29	3.7
7	Unspecified maternal hypertension	29	3.7
8	Maternal care for malpresentation of fetus	20	2.6
9	Other obstructed labour	13	1.7
10	Abnormalities of forces of labour	13	1.7
11	Maternal diseases unclassified	13	1.7
12	Obstructed labour due to malposition and malpresentation of fetus	11	1.4
13	Other complications (pyrexia)	10	1.3
14	Maternal care for disproportion	10	1.3
15	Antepartum haemorrhage	9	1.2
16	Other disorders of amniotic fluid	7	0.9
17	Venous complications and haemorrhoids in pregnancy	6	0.8
18	Multiple gestation	6	0.8
19	Maternal infectious and parasitic diseases ( viral hepatitis)	6	0.8
20	Diabetes mellitus in pregnancy	6	0.8
21	Placenta praevia	4	0.5
22	Pre-eclampsia	4	0.5
23	Gestational oedema and proteinuria without hypertension	3	0.4
24	Preterm labour and delivery	2	0.3
25	Complications specific to multiple gestation	2	0.3
26	Post-partum haemorrhage	2	0.3
27	Other obstetric trauma (Ruptured uterus)	1	0.1
28	Excessive vomiting in pregnancy	1	0.1
29	Maternal care for known or suspected fetal abnormality (microcephaly)	1	0.1
30	Single delivery by caesarean section	1	0,1
31	Polyhydramnios	1	0,1
32	Failed induction of labour	1	0,1
33	Unclassified reasons	162	20,8

**Means of transport, conditions of transport and staff accompanying women to hospital:** a total of 322 (64.9%) women were brought to our maternity centre in an ambulance, 173 (34.9%) in private cars, taxis or other readily available modes of transportation and only one woman (0.2%) was transported by Urgent Medical Aid Service (SAMU). During transfer, 320 (64.7%) were accompanied by a member of the staff from the referring facility (nurse, midwife or doctor). One hundred and seventy one (35.5%) were not accompanied by any member of the staff from the referring facility, but rather by a member of their family (Three women were accompanied together by a nurse and a member of their families). Only 13 women (2.6%) required intravenous infusion during transfer. The median of the distance travelled during the transfer was 49km (range 4-179km). When it was specified (292), the transfer was indicated in 62.3% of cases by a midwife and by a doctor in the remaining cases. Half of patients (249) were referred during the first stage of labour (Table 2).

**Table 2:** Means of transport, conditions of transport and staff accompanying women to hospital

		N	(%)
**Referral Maternity**	Enfidha	149	29,9
	KalâaKbira	105	21,0
	Msaken	99	19,8
	Bouficha	37	7,4
	other governorate	109	21,8
**Means of transport**	Ambulance	322	64,9
	Personal car	106	21,4
	Taxis	38	7,7
	Public Transport	29	5,8
	Urgent Medical Aid	1	0,2
	Service		
**Staff accompanying women to hospital**	Nurse	280	56,6
	Family	174	35,5
	Midwife	38	7,7
	Doctor	2	0,4
**Transfer indicated by**	Doctor	110	22,1
	midwife	182	36,5
	Unspecified	206	41,4
**Stage of labour**	Not in Labour	151	30,2
	In Labour	313	62,6
	Immediate post-partum	4	0,8
	Unspecified	32	6,4
**Reference maternity Warned**	Yes	9	1,8
	No	491	98,2
**Documents provided by the referral maternity**	Transfer sheet	400	80,0
	Partograph	228	45,8
	Fœtal heart rate	195	39,2
	Referral letter	83	16,6
	none	10	2,0

**Waiting times:** the average length of stay in the referring maternity before transfer was 40 min (range 0 to 1815 min). After arrival to the receiving maternity, the average wait time before being seen by an obstetrician or a midwife was 5 min (range from 0 to 110 min). The first exam in the receiving maternity was done by a midwife in 97.4% of cases.

**Information provided by referring facilities:** prior to transfer, the receiving maternity was informed of the referral only in nine cases (1.8%). The majority of referred women (400 (80%)) attended the receiving maternity with a referral letter. Two hundred and twenty eight women (45.8%) came with pantographs; this was 77.2% of the 176 women referred while in labour period.

**Pertinent findings on arrival to the receiving maternity:** the patient's general conditions waere assessed on arrival to the receiving maternity to be preserved (good) in 420 (98.8%) women. Preserved condition described women who were not perceived to have a life threatening condition. The most frequent indications for referral in those in poor condition were haemorrhagic shock, unstable hemodynamic status, hypertension, and severe anaemia. Among women referred, before delivery, foetal presentation was vertex in 452 (93.7%), breech in 14 (2.9%), transverse in one (0.2%) and face in one (0.2%). Membranes were intact in 243 (50.2%) of cases. The cervix was at least 4cm dilated in 295 (60%) of cases.

**Diagnoses:** twenty eight diagnoses were retained after referral with the most common diagnoses being prolonged pregnancy (29.5%) and premature rupture of membranes (PRM) (19.3%) (Table 3). It was noticed that 15% of PRM and 78% of prolonged pregnancy had not been diagnosed in peripheral maternities before transfer. The reasons for referral did not conform to the diagnoses established upon admission to our center in 41% of women and were almost entirely unjustified by the National Perinatality Programme.

**Table 3:** Diagnoses at the admission in the receiving maternity

	*Admission diagnosis*	*N*	*%*
1	Prolonged pregnancy	198	29.5
2	Premature rupture of membranes	130	19.3
3	Maternal care for abnormality of pelvic organs	69	10.3
4	Labour and delivery complicated by fetal stress	66	9.8
5	Maternal care for other known or suspected fetal problems	46	6.8
6	Maternal care for malpresentation of fetus	31	4.6
7	Gestational hypertension	22	3.3
8	Abnormal findings on antenatal screening of mother	20	3.0
9	Preterm labour and delivery	13	1.9
10	Diabetes mellitus in pregnancy	9	1.3
11	Pre-eclampsia	6	0.9
12	Multiple gestation	6	0.9
13	Ante-partum haemorrhage	6	0.9
14	Maternal infectious and parasitic diseases ( viral hepatitis)	6	0.9
15	Other complications : pyrexia during labour	6	0.9
16	Maternal care for disproportion	5	0.7
17	Venous complications and haemorrhoids in pregnancy	4	0.6
18	Obstructed labour	3	0.4
19	Post-partum haemorrhage	3	0.4
20	False Labour	2	0.3
21	Disorders of amniotic fluid (oligoamnios,…)	2	0.3
22	Pre-existant hypertension	2	0.3
23	Placenta praevia	2	0.3
24	Other obstetric trauma (Ruptured uterus)	1	0.1
25	Complications specific to multiple gestation	1	0.1
26	Perineal laceration during delivery	1	0.1
27	Premature separation of placenta (abruptio placentae)	1	0.1
28	Other maternal diseases classifiable elsewhere	11	1.6

**Treatment:** a total of 358 referred women (72.5%) had vaginal deliveries and 102 (20.6%) had caesarean section deliveries. Foetal distress was the main indication for caesarean section (31.6%). The median time to delivery was 4.25 hours. The shortest time-to-delivery was 0 min and the longest time 4.25 days.

**Outcomes:** one hundred and thirteen (22.9%) women were admitted to the recovery room, especially after caesarean section and 32 women were admitted to the intensive care unit (ICU) for hypertension related complications or haemorrhage. No maternal deaths were reported among referred women during the study period. Regarding fetal outcomes, 98.2% of babies were born alive and 1.8% were still born. A total of 46 (9.3%) newborn infants required admission to the neonatal ICU. The main indication for such admission was complicated fetal maternal infection (37%).

## Discussion

According to the 2013 regional statistics, 1539 pregnant women, out of 2092 obstetric admissions in peripheral maternities in the region of Sousse, were referred to FH maternity, which represent 73.5% [11]. In this study, obstetric admissions represent 15.23% of the obstetric activity in this facility. The maternal referral rates from peripheral maternities to central maternities reported in the literature vary widely and may reflect differences in health facilities [12-15], distance travelled during transport and different clinical criteria used for admitting and transferring women. Moreover, there is no standardized classification of the reasons for maternal transfers and for the admission and used transfer guidelines. For these reasons, a comparative analysis remains limited [16]. However, we can notice that in spite of the improvement of peripheral facilities in Sousse such as the creation of an operating room at the Regional Hospital of M'saken and the assignment of a specialist in obstetric in this facility, the transfer rate remains high, explaining the congestion and the overload in the FH maternity. On the other hand, according to the scoping review of the literature of Murray and Pearson about maternal referral systems in developing countries, concentration of almost all births into “referral level” hospitals does not guarantee a low maternity mortality ratio. In fact, overcrowding and poor technical ability result in poor quality of care [3].

The two most common reasons for referral in this survey were premature rupture of membranes and fetal distress. This finding was similar to those found in developed countries, mostly due factors related to labor, whereas it seems different from those founded in other African countries where the predominant reasons for referral were pregnancy-related complications and which could be identified during prenatal follow up. Indeed, according to studies carried out in Sub-Saharan Africa, transfer patterns were dominated by dystocia, hemorrhage, and also hypertensive complications, suggesting a lack of qualified staff in peripheral facilities [17] and the poor quality of antenatal follow-up [18]. On the other hand, studies carried out in developed countries, have cited a wide variety of reasons for transfer. For example, in the Netherlands, New Zealand and the United Kingdom, transfers were justified by prolonged labor, failure to progress during labor or fetal distress [13, 19-21]. Whereas in Australia [12] and France [22], the most frequent reasons were premature rupture of the membranes and preterm labor or delivery.

The transfer patterns noted in Tunisia may be attributed to the substantial improvement made in prenatal care. In fact, prenatal care, mainly in terms of the number of antenatal visits is adequate (at least 4 visits) for about 90% of cases in Tunisia [23]. In our study it was estimated at 81.6%, but it should not be forgotten that, a good quality of health care also depends on the temporal distribution and the content of such antenatal visits. We reported that in 95.8% of cases, reasons for referral did not conform to the National Perinatality Programme guidelines. On the one hand, this fact can be accounted for by the fact that midwives in peripheral maternities often have an over-cautious attitude and refer women even with the slightest problems to tertiary maternity centers. The fear of blame by patients' families or health authorities in case of unfavorable outcomes may be the main causes of this abuse of transfer. On the other hand, it should also be pointed out that the guidelines of this programme have been revised since 1990. According to Khalfaoui *et al.* [24], almost two-thirds of the interviewed Tunisian midwives felt that this program was well designed (65.8%) and it provided sufficient guidance about the conditions of maternal transfer. However, for 44.73% of them, some indications for transfer deserve to be reviewed.

In this context, providers need protocols to guide them in determining at what point in the course of a complication or at what level of risk, they should refer a woman to higher level of care [25]. Such referral guidelines need to reflect local epidemiological conditions, organisational capacity, and community preferences [3]. Compared to poorly resourced systems, referral transportation in this study can be considered less disturbing. In fact, 64.9% of women referred to our center were brought in by ambulance and 64.7% were accompanied by a staff member from the referring facility. But travelling a distance of 49km (range 4-179km) when the referral is unnecessary can be unsafe for the both the mother and the foetus especially if any means of transportation other than an ambulance is utilized. The time data reported in this study were mainly obtained from women interviewed; rare were cases when these informations were noted in the transfer letter or in the medical record. Time is often of crucial importance in obstetric emergencies. The “three delays” model is a stepwise model that attempts to analyse reasons for postponement of treatment: decision-making, accessing services and receipt of appropriate care once a health facility is reached. The third delay, although under-researched, is likely to be a source of considerable inequity in access to emergency obstetric care in developing countries [26]. It is disturbing that the receiving maternity was informed of the referral in only nine cases of referrals. This fact highlights the lack of adequate communication between different levels of the referral system, which can negatively impact care provision.

The main means of communication was the referral letter (80%). According to the review of Murray and Pearson, referral communications have to relay increasingly on sophisticated technologies such as the use of telemedicine to make referrals more appropriate [3]. Moreover, more than 70% of the women referred during the labour period came with partograph which had been a valuable tool for tracking the progress of labour, making decisions as regards the well being of the mother and the foetus. However, although it has been successfully integrated into routine practice, the link between “use”, decision making, and successful referral action can still require attention [27]. The reasons for referral did not conform to the diagnoses established on admission in 41% of the referred women. This fact can result in the congestion and the overcrowding of the central maternity and a significant increase in workloads in addition to financial costs for the patient and her family. However, seeking more diagnostic precision before referring can lead to delayed transfers [7, 28]. Besides these unnecessary referrals, pathologic cases of women may go unnoticed. In fact, in this study, cases of post partum haemorrhage, retro-placental hematoma, 15% of PMR and 78% of prolonged pregnancy had not been diagnosed before referral. The median time to delivery was 4.25 hours. In an Australian study, this time was estimated to 24.4h. Forty three percent of women were delivered within 24 hours of admission and 29% were either delivered after 7 days or delivered elsewhere [12]. This variability can depend on the reason for transfer.

Finally, no maternal deaths were reported among referred women during the period of study and only 1.8% babies were still born. This fact highlights the effectiveness of the health care system in terms of outcomes (maternal and neonatal mortality indicators). However, there are still a few challenges that need to be addressed to improve the quality of care delivered to pregnant women. This study is among the rare Tunisian studies to document obstetric referrals to a Tunisian tertiary level maternity and to assess the conformity of referral mechanisms with the Tunisian National Perinatality Programme guidelines. In this context, the adequacy of the transfer must be considered among the process' indicators which must be regularly estimated in the same way as outcome indicators such as maternal mortality rate. Also, in this study data was obtained from a large cohort of women referred to a single tertiary care maternity and were collected prospectively from various sources: medical records, referral letters, and interviews with referred women and the receiving midwife. This fact can limit bias resulting from the missing data in medical records, but there were limitations when we had to extract reasons for referral or diagnoses for admission from medical records. No standard classification was used in records. Coding these reasons and diagnoses according to The International Statistical Classification of Diseases (ICD-10) and related health problems was time consuming and was beyond the scope of this observational study.

## Conclusion

To improve the existing referral system in the region of Sousse, serious steps should be taken to address the current challenges. In fact, having an adequate referral center and an accepted referral transportation are not sufficient for an effective referral system. This will later require a communication and feedback system, a consensus on specific protocols for the identification of complications, trained personnel in their use, teamwork between referral levels, a unified records system and mechanisms to ensure that patients do not bypass a level of the referral system.

### What is known about this topic

- Improving maternal health was a worldwide priority for the global health and development community;
- The process of access to care system and especially the referral system is a key dimension in saving mothers' lives;
- For more effective planning medical and community-based actions to improve maternal health, an inventory of this system of referral is necessary.

### What this study adds

- There is no standardized classification of the reasons for maternal transfers and for the admission and used transfer guidelines;
- The two commonest reasons for referral were premature rupture of membranes and fetal distress;
- In most reported cases, reasons for referral did not conform to the national perinatality programme.

## Competing interests

The authors declare no competing interests.
